# Adaptability and Resilience in Aging Adults (ARIAA): protocol for a pilot and feasibility study in chronic low back pain

**DOI:** 10.1186/s40814-021-00923-y

**Published:** 2021-10-19

**Authors:** Paige E. Lysne, Shreela Palit, Calia A. Morais, Lucas C. DeMonte, Maria Lakdawala, Kimberly T. Sibille, Emily J. Bartley

**Affiliations:** 1 Department of Community Dentistry and Behavioral Science, Pain Research and Intervention Center of Excellence, University of Florida, Gainesville, FL USA; 2 Department of Counseling and Higher Education, Northern Illinois University, DeKalb, IL USA; 3 Department of Microbiology and Cell Science, University of Florida, Gainesville, FL USA; 4 Department of Aging and Geriatric Research, Pain Research and Intervention Center of Excellence, University of Florida, Gainesville, FL USA

**Keywords:** Resilience, Low back pain, Aging, Positive psychology, Positive affect, Pain acceptance, Hope, Pain self-efficacy, Quality of life, Disability

## Abstract

**Background:**

Chronic low back pain (cLBP) is the leading cause of disability among older adults and one of the top reasons for seeking healthcare, resulting in significant decrements in physical functioning. Because older adults are among the fastest growing cohorts in the USA, both the incidence and burden of cLBP are expected to increase considerably, rendering geriatric pain management a top health priority. Resilience is defined as a process allowing individuals to adapt and recover from adverse and stressful conditions, and it has been highlighted as a crucial factor in positive health-related functioning. While a growing body of literature supports the use of resilience-based interventions in chronic pain, research examining their effectiveness in older adults with cLBP remains limited. The primary aims of the study are to assess the feasibility and acceptability of a psychologically oriented resilience intervention among aging adults with cLBP.

**Methods:**

In this article, we describe the rationale and design of the Adaptability and Resilience in Aging Adults (ARIAA) study, a single-arm intervention in which 60 participants (ages ≥ 60 years) with cLBP will be recruited to participate in a 7-week group-based program aimed at enhancing psychological resilience. Intervention sessions will target positive psychology concepts (e.g., positive affect, pain acceptance, hopeful thinking, pain self-efficacy) and cognitive behavioral techniques that have established benefits in pain management. Primary study outcomes include intervention feasibility and acceptability as measured by treatment engagement, intervention credibility and satisfaction, ability to meet recruitment and retention metrics, and the feasibility of questionnaire and home activity completion. Outcomes will be assessed at baseline, immediately at posttreatment, and at the 3-month follow-up period.

**Discussion:**

This study will establish the feasibility and acceptability of a novel intervention aimed at enhancing positive, psychological functioning, and resilience in older adults with cLBP. Achievement of these aims will provide a rich platform for future intervention research targeting improvements in pain and disability among geriatric populations and will serve as a foundation for a fully powered trial to examine treatment efficacy of the proposed intervention.

**Trial registration:**

Clinicaltrials.gov, identifier NCT04068922. Registered 28 August 2019.

**Supplementary Information:**

The online version contains supplementary material available at 10.1186/s40814-021-00923-y.

## Background

Chronic low back pain (cLBP) is the leading cause of disability worldwide [1] and represents the most therapeutically challenging pain condition among older adults [2, 3], affecting approximately 36% of adults over the age of 60 years [4]. cLBP is associated with impairments in psychological, cognitive, and physical functioning and is one of the top reasons for seeking healthcare [5–7]. Evidence suggests that the prevalence of disabling back pain increases with age [8], thus adding to its tremendous disease burden. Because older adults are among the fastest growing cohorts in the USA, both the incidence and burden of cLBP is expected to increase considerably. Despite this, pain management is frequently suboptimal among older adults. Indeed, pharmacological therapies show limited clinical efficacy and a greater risk of side effects, multimorbidities common to geriatric populations complicate pharmacological treatment, and polypharmacy can result in adverse drug reactions [2]. As such, the demand for safe and effective therapeutic targets to reduce pain and associated functional limitations in later life is of critical importance.

Research has been historically focused on vulnerability and risk factors (e.g., negative mood, pain catastrophizing, fear avoidance) influencing pain and functioning. This represents a significant knowledge gap as emerging literature suggests that individuals with chronic pain have the ability to exhibit resilience [9–14]. Conceptualized as a dynamic construct, resilience is defined as a process of adapting to adversity, threats, or significant sources of stress [15]. Individuals with greater levels of resilience are known to persist in meaningful activities despite ongoing hardship, quickly rebound from physical or emotional stress, and experience personal growth as a result of adversity [16]. While factors such as anxiety and depression may increase risk for pain vulnerability, psychosocial facets such as optimism, self-efficacy, gratitude, and positive emotions are known to promote greater pain-related resilience and improve quality of life [16–19]. Existing research [9, 10, 20, 21] signifies that resilience (and its underlying facets) is associated with attenuated experimental pain sensitivity [22]; lower pain severity, disability, and mobility impairment [9, 10, 23]; and higher psychological functioning [24]. Given this, there has been a growing body of evidence and increasing interest in better understanding resilience as a clinical target for older adults with chronic pain [16, 21, 25].

Recent years have witnessed a burgeoning interest in promoting adaptive functioning in individuals with chronic pain through interventions that capitalize on individual strengths and positive psychological resources. Embedded within positive psychology, strength-based approaches such as these offer advantages to conventional interventions (e.g., cognitive behavioral therapy). Specifically, existing treatments predominantly target the reduction of negative symptoms (e.g., reduction in maladaptive thoughts and sleep impairment), with evidence suggesting only modest effects on pain and disability that are not consistently maintained over time [26–29]. Hence, existing strategies are not sufficient.

Therapeutic approaches aimed at fostering resilience may be optimally positioned to address these limitations and provide symptomatic improvement in individuals with chronic pain. For instance, improvements in pain severity and interference, psychosocial function, and experimental pain sensitivity have been observed from interventions targeting humor, social support, and hope [30–32]. Further, a growing body of literature supports the use of positive activity interventions (i.e., activities aimed at boosting positive emotions, cognitions, and behaviors such as gratitude building and pleasant activity scheduling) in individuals with low back pain [33], chronic pain secondary to physical disability (i.e., spinal cord injury, multiple sclerosis, neuromuscular disease, and postpolio syndrome) [34, 35], bodily pain [36], osteoarthritis [37], and musculoskeletal pain [38] with small to large effects observed in a number of outcomes including mood, physical and psychosocial function, clinical pain severity, and well-being. Together, these findings align with the Broaden-and-Build theory [17] which signifies that repeated experiences of positive emotions broaden people’s attentional focus and behavioral capabilities [39] to facilitate the building of resources [40] that optimize physical and psychological functioning [41, 42]. In the context of chronic pain, this could suggest that positive affect experienced during behavioral engagement may increase non-conscious drive for that activity, thereby facilitating adherence to positive health behaviors that optimize pain management. Although evidence suggests that increasing positive emotions may be a promising target for pain intervention, a significant knowledge gap remains as to whether results can be translated to older adults with cLBP given the lack of existing research in this cohort.

Following the National Institutes of Health (NIH) Stage Model guidelines for the development and preliminary testing of new behavioral therapies [43], this Stage I pilot study will examine the feasibility and acceptability of a 7-session group-based resilience intervention (i.e., Adaptability and Resilience in Aging Adults (ARIAA)) for older adults with cLBP. Intervention modules will incorporate concepts previously shown to support adaptive health-related outcomes (e.g., positive affect, pain acceptance, hopeful thinking, pain self-efficacy) while also drawing upon additional cognitive behavioral techniques (i.e., relaxation, mindfulness, cognitive reappraisal) with known benefits for pain management. As an exploratory aim, we will examine whether improvements in pain intensity, pain interference, negative mood, and quality of life are predicted by changes in proposed study mediators (i.e., positive affect, pain acceptance, hope, pain self-efficacy).

## Methods

### Design overview

**Fig. 1:**
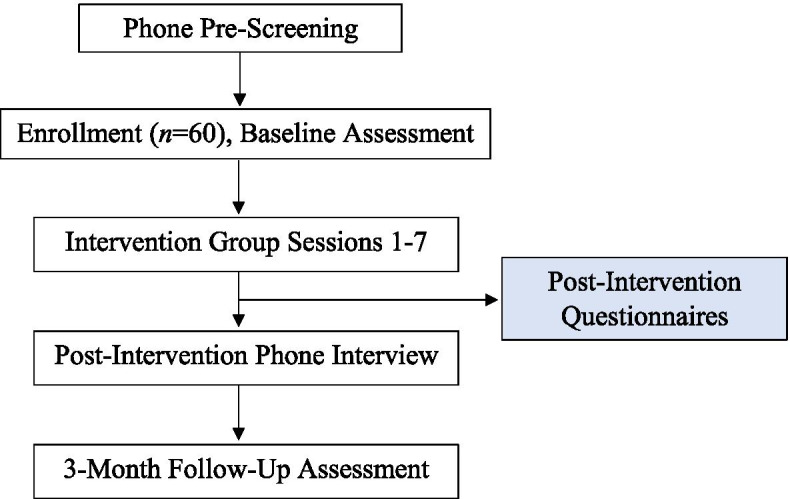
Flowchart of study procedures

This single-arm trial funded by the National Institute on Aging/National Institutes of Health (R00AG052642) will examine the feasibility and acceptability of a psychological resilience intervention for 60 older adults (ages ≥ 60 years) with cLBP. The intervention will include 7 weekly group sessions (1.5 h each) targeting content adapted from traditional cognitive behavioral therapy for pain (CBT), acceptance and commitment therapy (ACT), and positive psychology concepts focused primarily on increasing positive affect, pain acceptance, hope, and self-efficacy. Data will be collected at baseline, on completion of the 7-week treatment, and at 3 months posttreatment (3 months after the final intervention session). If the trial is found to be feasible, the study findings will inform the design of a full-scale randomized clinical trial. The flow of procedures has been outlined in Fig. 1.

### Specific aims

The primary aims of this trial are to examine the acceptability of the intervention, the feasibility of recruitment and adherence to the intervention, and preliminary treatment effects. We operationalized the concepts of acceptability, feasibility, and efficacy as follows:

- *Specific aim 1*: Evaluate the acceptability of the intervention, as indicated by treatment engagement and participant reported treatment credibility and satisfaction.
- *Specific aim 2*: Assess intervention feasibility, including the ability to meet recruitment and retention metrics, and the feasibility of questionnaire and home activity completion*.*
- *Exploratory aim*: Determine the extent to which intervention-related improvements in pain intensity, pain interference, negative mood, and quality of life are predicted by changes in four primary mechanism variables: positive affect, pain acceptance, hope, and pain self-efficacy*.*

### Study setting and recruitment

This single-site study will be conducted at the University of Florida (UF) in Gainesville, FL, USA. Sessions will be held in a conference room with comfortable seating and access to whiteboards and a computer screen for demonstration of key intervention concepts and activities. Participants will be provided a parking voucher upon their departure. Due to COVID-19, intervention sessions may be delivered online through PHI Zoom.

Participants will be recruited via self-referral through a variety of clinic and community-based resources throughout the larger Gainesville, FL community including provider referral, study flyers placed in primary and secondary care pain clinics as well as around the University of Florida campus and health clinics, radio and newspaper advertisement, health fairs, and social media. We will also recruit via targeted mailings to individuals in our UF Pain Research and Intervention Center of Excellence (PRICE) registry (i.e., registry of > 1000 adults with pain who have provided consent to be contacted for future pain research) and the UF Clinical and Translational Science Institute (CTSI) HealthStreet registry (i.e., a service that is dedicated to recruiting underserved and minority populations within the Gainesville, FL community). Prospective participants will have the opportunity to contact the study team if interested and complete initial screening eligibility via telephone or in person prior to enrollment.

### Participants

A sample of 60 participants (to achieve the goal of 50 participants who complete the study; see “Statistical analysis” below), ages 60 and older, will be invited to participate. Study inclusion criteria are as follows: (1) endorsement of pain in the lower back region (i.e., space between the lower posterior margin of the rib cage and the horizontal gluteal fold [44], (2) back pain reported of moderate or severe intensity (rating of ≥ 3 on a numeric rating scale ranging from 0 to 10), (3) back pain occurring on at minimum half of the days in the past 6 months [44]; and (4) back pain moderately interferes with daily activities (rating of ≥ 3 on a numeric rating scale ranging from 0 to 10). Given the comorbidity with other pain conditions [45, 46] and to generalize results more broadly, participants with other musculoskeletal conditions will also be eligible as long as low back pain is identified as the primary pain complaint.

Participants will be excluded for the following: (1) current participation in another psychological treatment, (2) severe psychiatric illness not adequately controlled by medication (e.g., schizophrenia, bipolar disorder) or other conditions anticipated to impair intervention engagement (e.g., substance abuse/dependence), (3) presence of chronic, malignant pain (e.g., HIV, cancer) or systemic inflammatory disease (e.g., rheumatoid arthritis, ankylosing spondylitis, systemic lupus erythematosus, etc.), (4) significant cognitive impairment on the Montreal Cognitive Assessment [47], (5) inability to read and write English, (6) back surgery within the past 6 months or future surgeries scheduled within the study time frame, and (7) if currently taking prescription analgesic or psychotropic medication, must be stabilized on these treatments for ≥ 4 weeks prior to the baseline assessment. Exclusion criteria were selected to ensure that participants are not at increased risk for discomfort or harm and to protect the integrity of the data due to medical comorbidities that may impact study findings.

### Screening and enrollment

All study procedures (Table 1), including screening and enrollment, will be conducted by trained personnel with backgrounds in psychology and public health. All interested participants will first undergo a brief screening interview, via telephone or in person, to determine eligibility. Participants will be queried on their self-reported age and health history including the presence of major medical illnesses, recent back-related injuries or surgeries, and low back pain symptoms to ensure that no exclusion criteria are present. If eligible, participants will be invited to participate in a 1.5-h baseline assessment for a more thorough evaluation of eligibility.

**Table 1:** Study procedures

	Prescreen	Screening (baseline)	Intervention sessions							Post	3 months
		Visit 1	Visit 2	Visit 3	Visit 4	Visit 5	Visit 6	Visit 7	Visit 8		
**Procedures**											
Contact information	X										
Prescreening interview	X										
Eligibility review	X	X									
Informed consent		X									
Demographic information		X									
Medical history review		X	X	X	X	X	X	X	X		
Adverse event review			X	X	X	X	X	X	X		
Cognitive assessment (MoCA)		X									
**Measures of treatment acceptability and feasibility**											
Treatment engagement (Therapist)			X	X	X	X	X	X	X		
Treatment engagement (Participant)			X	X	X	X	X	X	X		
Treatment expectancy		X	X								
Treatment module evaluation									X		
Treatment evaluation (Qualitative)										X	
Global treatment satisfaction									X		
Home activities evaluation				X	X	X	X	X	X		
**Treatment outcomes and mediators**											
PROMIS Pain Intensity		X							X		X
PROMIS Pain Interference		X							X		X
PROMIS Mood (Dep, Anx)		X							X		X
WHOQOL-BREF		X							X		X
PANAS		X							X		X
CPAQ		X							X		X
ASHS		X							X		X
PSEQ		X							X		X

*PROMIS* Patient-Reported Outcomes Measurement Information System, *Dep* Depression, *Anx* Anxiety, *WHOQOL-BREF* World Health Organization Quality of Life–Brief, *PANAS* Positive and Negative Affect Schedule, *CPAQ* Chronic Pain Acceptance Questionnaire, *ASHS* Adult State Hope Scale, *PSEQ* Pain Self-Efficacy Questionnaire

During the baseline assessment, informed consent will be reviewed by the study PI or staff including a description and timing of the study procedures, potential risks and benefits of study involvement, rights to withdraw from the study, and details regarding protections against study risks. All participants will receive a copy of their signed informed consent form. After the consent process, participants will complete a demographic and medical history questionnaire assessing sociodemographic characteristics, reported duration of cLBP, comorbid medical conditions, and current medication use. Participants will also complete psychological and pain-related questionnaires assessing study-relevant outcomes and complete the Montreal Cognitive Assessment (MoCA) as an evaluation of cognitive functioning [47]. The MoCA is a widely used screening assessment for detecting cognitive impairment. Participants with scores < 26 will be excluded as this could interfere with their engagement in the study intervention. At the completion of the baseline assessment, eligible participants will be scheduled for their first intervention session approximately 1 week later. Participants will be informed that they may continue to receive their usual medical care.

### Study intervention

Participants will be offered seven 1.5-h group intervention sessions scheduled once weekly for 7 weeks. This format was selected based upon the structure of prior clinical trials [48, 49] and evidence that intervention effects on pain and psychological outcomes in older adults are strongest when delivered using group-based approaches (relative to an individual format) [28]. Approximately 6 to 8 individuals will participate in each intervention group. To standardize the application of the intervention and to ensure treatment fidelity, the intervention will be manualized and will include interventionist and participant workbooks with materials (e.g., meditation CD) and handouts for discussion and home practice. Study intervention materials and workbooks were written by the study investigators and interventionists and adapted for an older adult population with cLBP (e.g., age-appropriate content and examples specific to cLBP, incorporation of larger font size on written materials). Participant workbooks were printed in color and written at the 6th grade reading level as measured by Flesh-Kinkaid Grade Level estimates. Participants who miss a group visit will be allowed to complete an individual makeup session, with the stipulation that only one missed group session will be permitted (to ensure the benefits of the group dynamic).

A full description of the resilience activities and content in the intervention program are summarized below and in Table 2. The Broaden-and-Build Theory and Social Cognitive Theory contribute to the conceptual framework underlying the intervention, as these theories have applicability to pain management and adaptive health maintenance [19, 50, 51]. Intervention content was derived from the PI’s (EJB) preliminary work establishing that positive affect, pain acceptance, hopeful thinking, and pain self-efficacy demonstrated the strongest effects (medium to large effects) on pain, disability, and emotional function among 60 older adults with cLBP (K99AG052642). Further, responses garnered during a series of focus groups and key informant interviews with the targeted population informed the development of/modifications to the intervention modules and activities. Briefly, participants reviewed the existing intervention manual for this initial phase of the study and provided feedback on the usefulness of the resilience modules (including any new content to be added), and preferences regarding delivery frequency (e.g., weekly, biweekly) and mode of delivery (e.g., individual, group). Discussions were moderated and co-moderated by research team members with training and experience in conducting focus groups. A range of positive psychology, CBT, and ACT techniques will be incorporated in the intervention to enhance pain resiliency (i.e., pleasant activity scheduling, value-driven behavior, pain acceptance, hopeful thinking and goal-directed behavior, positive events and reappraisal, and pain self-efficacy). All activities were selected due to strong empirical evidence supporting their use [52–55], ease of completion, and applicability to the targeted resilience constructs.

**Table 2:** Overview of resilience intervention session content

**Session**	**Content**	**Home Practice**
WK 1: introduction	Symptoms and causes of back pain. Rationale for resilience in managing pain.	Complete a character strength survey. Record personal strengths.
WK 2: pleasant activities	Benefits of gratitude, pleasant activities, and pacing in the context of pain.	Complete a log of gratitude practice. Engage in weekly pleasant activities.
WK 3: values-based living	Values-based activity and mindfulness practice for pain.	Complete a values assessment. Practice mindfulness exercises.
WK 4: hopeful thinking	Setting and achieving attainable goals. Using personal strengths to reach goals.	Select a goal to achieve. Identify areas in life that inspire hope.
WK 5: positive reappraisal	Interpreting stressful events and learning ways to reframe negative situations.	Log positive events and situations where reframing was helpful.
WK 6: self-efficacy	Discuss methods to enhance self-efficacy. Education on relaxation for stress and pain.	Practice diaphragmatic breathing exercise and record stress/pain levels.
WK 7: review	Review of skills learned during group. Feedback on group activities and skills.	Continue to practice resilience skills after the end of group.

Each session will follow the same format including a review of the previous week’s content (e.g., home activity review), barriers to home activity completion, an overview and discussion of the session objectives including didactic in-session activities, and assignment of home activities. A photocopy of each participant’s home activity will be made at the beginning of each intervention session and will be reviewed solely by the study PI and intervention facilitators after each session to assess quantity and quality of completion.

### Intervention content

#### Week 1: introduction and background

Participants will be provided with instruction on group intervention guidelines (e.g., confidentiality, respect, privacy), an overview of the purpose and structure of the program, education on cLBP etiology and symptoms and the Gate Control Theory of Pain [56], and the rationale for using resilience-enhancing activities for the management of cLBP. A discussion of the Upward and Downward Spirals of Emotions [50, 57] with respect to pain will be provided, as well as the use of character strengths [58] to boost resilience. As a home exercise, participants will be asked to complete the Values in Action (VIA) character strengths online survey to identify individual core virtues and positive attributes [59, 60] and to make note of one occurrence of using a personal strength during the next week.

#### Week 2: pleasant activities

Participants will be given instruction on how to increase the number of pleasant events in their life and will be provided with a list of pleasant activity ideas [61]. Education on the benefits of activity pacing will be discussed including ways in which to modify activity engagement to reduce pain flare-ups. The purpose of gratitude will be reviewed, and participants will be asked to identify elements in their life that they are thankful for and appreciate. For their home practice, participants will be encouraged to engage in three pleasant activities over the week and to record events/aspects they are grateful for each day.

#### Week 3: pain acceptance

ACT techniques will be used to help participants identify their personal values and to recognize the importance of living a meaningful and goal-directed life aligned with those values despite pain. A values assessment will be administered during the group session to assist participants in recognizing important values in their life [62]. Participants will also receive education and training in mindfulness meditation and will participate in a guided mindfulness exercise (i.e., Leaves on a Stream) [63] during the group session. A CD will be provided for at-home practice and participants will be encouraged to practice mindfulness exercises for a minimum of three times over the course of the week using pre-recorded scripts of Leaves on a Stream and a 5-min mindful breathing exercise (i.e., technique emphasizing focused attention on the breath).

#### Week 4: hope and goal setting

Based on Snyder’s theory of hope [55], participants will focus on their personal strengths and learn ways in which to create attainable goals (i.e., S.M.A.R.T. goals) [64] through the development of pathways (i.e., perceived ability to generate routes to goals) and agency (i.e., mobilization of efforts to achieve goals) thinking. Discussion will focus on making goals more specific, measurable, attainable, relevant, and time-bound and will target barriers and personal strengths that influence goal attainment. A scripted visualization exercise will be conducted during the session whereby participants will imagine themselves successfully reaching their goals. Participants will be asked to develop a goal they would like to achieve over the course of the intervention (using the S.M.A.R.T. goal framework) to facilitate hopeful thinking and goal-directed behavior and to identify areas in their life that inspire hope [32].

#### Week 5: positive events and reappraisal

To increase attentional focus on positive events, participants will be given instruction on how to identify negative or unhelpful automatic thoughts, evaluate automatic thoughts for accuracy, and downregulate negative emotions and thoughts through positive reappraisal [65]. Instruction will also be provided on prolonging and enhancing the experience/impact of positive events (e.g., telling someone about the event, replaying the event in your mind). As a home activity, participants will be encouraged to record one positive event that occurred every day and ways in which they strengthened that experience, as well as negative events in which they had to practice positive reappraisal [66].

#### Week 6: pain self-efficacy

While it is anticipated that self-efficacy will gradually be fostered throughout the course of the intervention (i.e., skills mastery experiences, feedback on progress, social persuasion through group support), this session will complement ongoing training by targeting problem-solving skills and confidence building, as well as empowering participants to promote adaptive pain management behaviors [1, 67]. The fear avoidance model of pain [68] will be introduced, and education will be provided on how fear can induce inactivity, thereby facilitating increased pain and disability. As a method of increasing self-efficacy in managing pain, a brief diaphragmatic breathing exercise will be practiced during the group session. Participants will be asked to engage in daily diaphragmatic breathing as a form of relaxation and to record levels of stress and pain before and after their practice (0–10 numeric rating scale).

#### Week 7: review of skills and wrap-up

Skills learned in the previous sessions will be reviewed and specific plans for the maintenance of resilience activities will be discussed. Participants will complete posttreatment outcome questionnaires and provide feedback on the intervention program.

### Intervention facilitators

Sessions will be administered by postdoctoral clinical psychology fellows who have experience administering psychological treatments for chronic pain. Intervention facilitators will be assigned in pairs to co-lead the study groups. Interventionists will be trained and supervised by the study investigators (EJB, KTS), each of whom has recognized expertise in delivering psychological interventions for individuals with chronic pain. Interventionists will receive extensive didactic and experiential training from the study PI. During training, interventionists will be assigned reading materials relevant to the study intervention [17, 69–72] and receive didactic and experiential training involving the delivery of each treatment session module in a mock group fashion.

### Treatment fidelity monitoring

Treatment fidelity will be monitored through several methods. First, each group session will be audio-recorded (with participant consent), and a random selection of 50% of these recordings will be reviewed by the study Co-I (KTS) to ensure that treatment procedures and content have been followed. Second, interventionists will review a portion of their audio-recorded group sessions for self-evaluation and improvement of skills. Third, interventionist compliance with the treatment protocol, appropriateness of the interventionist’s behavior, and quality of treatment delivery will be assessed using an adapted Cognitive Therapy Adherence and Competence Scale (CTACS) [73] that will be modified to align with the session content. Fourth, interventionists will be provided a structured therapist treatment manual to deliver the treatment sessions and will participate in weekly group supervision with the study PI involving corrective feedback and discussion of protocol fidelity, quality of intervention delivery, and group dynamics. And fifth, interventionists will be provided with a checklist of procedures specific to each session, and each checklist will be reviewed to ensure treatment fidelity.

### Participant retention

Several strategies will be implemented to maximize retention. At screening, participants will be educated on the importance of attending sessions. Participants will be sent frequent reminders of their appointments per our study contact protocol, and we will contact participants via a variety of modalities, including phone and email to make initial contact, or a mailed letter if unable to reach via phone or email. Participants who fail to attend their scheduled group session will be offered the opportunity to make this session up prior to attending the next session. To acknowledge the time and effort involved, financial payment will be provided for the completion of study procedures.

### Safety monitoring

For the purposes of monitoring safety, participants’ health status will be reviewed during each session including documentation of any changes in the amount or type of medications that they are currently taking, or the initiation of new therapies or treatments (e.g., counseling, acupuncture, physical therapy) for pain. The study PI will be alerted on any changes and/or adverse events reported by participants, and these will be recorded in progress notes and reported to the study physician for review, if necessary. All serious and unexpected adverse events will be reported to the study’s sponsor and the University of Florida IRB within 5 working days. Other types of adverse events will be monitored and reported to the University of Florida IRB in the study’s annual progress review. The investigators will review all reported adverse events and deviations on a weekly basis to minimize the risk to study subjects.

### Data handling and storage

Questionnaire data will be collected via a secure internet-based data collection system (i.e., REDCap) or on paper depending on participant preference. Responses derived via paper format will be transposed by study staff into REDCap. To ensure data accuracy, a secondary staff member will review all entries and compare them with the source data. Paper and computer records will be anonymized and identified only by subject number. Study records will be stored in locked file cabinets and will only be available to the PI or other project staff. Computer data files will be stored on computer servers with secure passwords, and electronic storage devices will be encrypted. Each intervention group session will be audio-recorded for the evaluation of interventionist compliance with the protocol. No identifying information will be transcribed as the purpose of this digital recording will be to assess treatment integrity. Once the audio recordings are reviewed and fidelity is discussed with study interventionists, the recordings will be destroyed. Prior to destruction, the recordings will be kept in a locked filing cabinet in the laboratory of the PI.

### Assessments and measures

As shown in Table 1, several questionnaires will be administered to assess general health, pain-related symptoms, and psychological functioning. In order to attenuate participant burden, questionnaires administered at intervention completion will be distributed either during the final intervention session or at home between the last two sessions (either via our secure internet-based data collection system (i.e., REDCap) or on paper).

### Intervention acceptability measures

#### Session-level engagement

Treatment engagement questions [74] will be adapted for the current study to assess participants’ effort exerted during group activities, completion of homework, and engagement in group discussions. This questionnaire consists of 5 items rated on a 9-point Likert scale ranging from 0 (none) to 8 (a lot), with two additional questions querying on the completion and quality of homework. Items will be completed by the study interventionists upon the completion of every group session for each attendee. A 6-item modified version of this scale will also be completed by each participant at the end of every session to assess their perceptions of engagement and interest in the session content and usefulness of the home activities.

#### Treatment credibility and expectancy

Treatment credibility questions [75] will be adapted for the current study as a treatment expectancy measure and administered during Visit 1 and Visit 2 consisting of the following 7 items: (1) reasonableness of the intervention, (2) willingness to undergo treatment, (3) confidence in recommending the intervention to others, (4) confidence that the intervention will help with pain coping, (5) confidence that the intervention will decrease pain, (6) confidence that the intervention will eliminate pain, and (7) expectation for improvement in pain symptoms.

#### Satisfaction with treatment module content

As a treatment evaluation and to better understand their perceptions of the intervention, participants will complete a study-developed questionnaire and be asked to indicate the usefulness of each intervention session module and home activity on a 5-point Likert scale ranging from 0 (not at all) to 4 (extremely). Approximately 1 week after the final intervention session, participants will also undergo a 20- to 30-min qualitative phone interview to obtain their views and experiences of the intervention. The protocol for the interviews will consist of a series of questions designed to elicit feedback regarding experiences with the intervention, usefulness of the intervention content and home activities, and perceived barriers to treatment engagement (see Additional file 1 for interview guide). Interviews will be facilitated by a clinical research coordinator and conducted individually. All interviews will be audio-recorded and transcribed by a professional transcriptionist.

#### Global treatment satisfaction

The 8-item Client Satisfaction Questionnaire (CSQ-8) [76] will be administered after the intervention as an assessment of treatment satisfaction. Items are rated on a 4-point Likert scale and measure aspects related to the quality of intervention, willingness to recommend the intervention, and general satisfaction with the intervention.

### Intervention feasibility measures

We will evaluate feasibility based on the ability to recruit, enroll, and retain participants, as well as adherence and completion of study questionnaires and home activities. Specifically, we will examine: (1) the number of individuals screened for eligibility, who were deemed eligible to participate and those who were enrolled in study procedures, including any reasons for ineligibility; (2) intervention completion, determined by the number of intervention sessions attended; (3) questionnaire feasibility including completion rates of treatment outcome and mediator measures (to determine which measures to carry forward to the full trial) and the degree of missing data present; and (4) feasibility of the home activities.

#### Home activities evaluation

Participants will complete a 7-item questionnaire that was adapted from Kazantzis et al. [77] for the purposes of assessing the utility of the home activities. Items are rated on a 7-point Likert scale ranging from 0 (not at all/none) to 6 (extremely/all). Three questions address feasibility (i.e., degree of home–activity completion, level of understanding regarding home activities, degree of time and effort needed to complete activities) and will be used in the analysis.

### Exploratory outcomes and mediator variables

Although this is not a traditional Stage II efficacy study, we will collect data on the following measures to provide preliminary data and to determine the feasibility of examining these constructs in a larger scale evaluation. Exploratory outcomes include pain intensity, pain interference, negative mood (i.e., depression, anxiety), and quality of life. Aligning with the NIH Science of Behavior Change approach [78], we will also evaluate the potential for intervention effects on the proposed resilience targets (i.e., positive affect, pain acceptance, hope, pain self-efficacy) and examine whether treatment-related changes in these variables are associated with improvements in pain outcomes.

#### Patient-Reported Outcomes Measurement Information System (PROMIS) Pain Intensity

The 3-item PROMIS Pain Intensity short form [79] evaluates average and worst back pain during the past 7 days, as well as pain at the time of questionnaire completion by providing a 1 (no pain) to 5 (very severe) pain rating. This scale has been used in patients with chronic pain and demonstrates excellent internal consistency (*α* = 0.91) [80].

#### PROMIS Pain Interference

The short form of the PROMIS Pain Interference measure [81] includes 8 questions (e.g., “How much did pain interfere with your day to day activities?”) examining pain-related impairment in social, cognitive, emotional, physical, and recreational activities over the past 7 days. Ratings are made from 1 (not at all) to 5 (very much), and higher scores indicate greater interference from pain. The pain interference scale demonstrates convergent validity with related measures and has excellent internal consistency (*α* = 0.99) [80].

#### PROMIS Emotional Distress Scales

The 8-item short forms of the PROMIS Depression Scale and the PROMIS Anxiety Scale [82] assess depressive (e.g., “I felt worthless”) and anxiety-related (e.g., “I felt fearful”) symptoms over the past 7 days. Respondents rate the frequency of their experience of each symptom from 1 (never) to 5 (always), with higher scores indicating a greater presence of symptomatology. Cronbach’s alpha demonstrates high internal consistency for both depression (*α* = 0.98) and anxiety (*α* = 0.97) scales [80].

#### World Health Organization Quality of Life–Brief (WHOQOL-BREF)

The WHOQOL-BREF [83] scale consists of 26 items assessing quality of life over the past week in four domains: physical health, psychological health, social relationships, and environment. The WHOQOL-BREF has good psychometric properties of reliability (*α* = 0.68 to 0.82 across domains) and performs well in tests of validity [83].

#### Positive and Negative Affect Schedule (PANAS)

The PANAS [84] is a 20-item scale assessing positive affect (PA) and negative affect (NA). Items are rated on a 5-point scale ranging from 1 (very slightly or not at all) to 5 (extremely), resulting in scale scores for PA and NA. Research supports the construct validity of the PANAS, with internal consistency estimates found to be high for both subscales: *α* = 0.89 for PA and 0.85 for NA [85].

#### Chronic Pain Acceptance Questionnaire (CPAQ)

The CPAQ [86] is comprised of 20 items measuring acceptance of pain. This measure has two subscales: activity engagement (i.e., pursuit of life activities regardless of pain) and pain willingness (i.e., disengaging from pain avoidance and control). Items are rated on a 7-point scale ranging from 0 (never true) to 6 (always true). The CPAQ shows moderate to high correlations with measures of avoidance, distress, and daily functioning and has good internal consistency, with alphas of 0.82 for activity engagement and 0.78 for pain willingness [86].

#### Adult State Hope Scale (ASHS)

The ASHS [87] includes 6 questions (e.g., “There are lots of ways around any problem that I am facing now”) that tap into state levels of hope. Two subscale scores assessing agency (i.e., sustained movement towards goals) and pathway (i.e., generation of workable routes to goals) thinking are derived, as well as a total score consisting of a sum of these two scales. Items are rated on an 8-point Likert scale ranging from 1 (definitely false) to 8 (definitely true). The ASHS demonstrates concurrent and discriminant validity, as well as good internal consistency estimates with alphas ranging from 0.82 to 0.95 over a 30-day period [87].

#### Pain Self-Efficacy Questionnaire (PSEQ)

The PSEQ [88] is a 10-item measure that examines pain-related self-efficacy. Scores range from 0 (not at all confident) to 6 (completely confident to undertake an activity), and higher scores indicate a greater level of self-efficacy in the midst of chronic pain. The PSEQ has excellent internal consistency (*α* = 0.92) and demonstrates significant correlations with measures of pain-related disability and coping [88].

### Statistical analysis

As this is a Stage I pilot feasibility study, a sample size calculation is not appropriate. However, our target sample size of 50 participants is consistent with prior recommendations [89–91] and will provide data on intervention acceptability and feasibility, as well as the potential to influence study outcomes and mechanisms of action. Accounting for a conservative 20% attrition rate, we will recruit an additional 10 participants (total *N* = 60). Because of the potential for high variability and imprecision in outcomes in pilot samples [89, 90, 92], differences across baseline and post-intervention measures will be analyzed using descriptive statistics with means, standard deviations, and ranges reported. In addition, 95% confidence intervals (CI) will be developed around each acceptability and feasibility mean. We will evaluate if predetermined metrics of success for each target fall within this interval. See Table 3 for a description of metrics that will support progression to a fully powered trial.

**Table 3:** Metrics for acceptability and feasibility outcomes

**Outcome**	**Major revision prior to full trial and monitor closely**	**Continue to full trial with modifications**	**Continue to full trial without modifications**
**Trial acceptability**			
Session-level engagement	° < 4 on a 9-point Likert scale, with 8 (0–8 range) representing the highest possible engagement.	° Score of 4 or 5 on a 9-point Likert scale.	° ≥ 6 on a 9-point Likert scale.
Treatment credibility and expectancy	° < 5 on an 11-point Likert scale, with 10 (0–10 range) representing the highest level of credibility.	° Score of 5 to 7 on an 11-point Likert scale.	° ≥ 8 on an 11-point Likert scale.
Satisfaction with intervention content	° < 2 on a 5-point Likert scale, with 4 (0–4 range) representing the highest degree of satisfaction with session content.	° Score of 2 on a 5-point Likert scale.	° ≥ 3 on a 5-point Likert scale.
Global treatment satisfaction	° < 2 on a 4-point Likert scale, with 4 (1–4 range) representing the highest level of global treatment satisfaction.	° Score of 2 on a 4-point Likert scale.	° ≥ 3 on a 4-point Likert scale.
**Trial feasibility**			
Enrollment rates	° < 70% of participants who enroll commence treatment.	° 70–90% of participants who enroll commence treatment.	° > 90% of participants who enroll commence treatment.
Participant retention	° < 70% participant retention by the 7-week time point.	° 70–80% participant retention by the 7-week time point.	° > 80% participant retention by the 7-week time point.
Questionnaire feasibility	° < 80% completion of study questionnaires (treatment outcomes and mediators).	° 80–90% completion of study questionnaires.	° > 90% completion of study questionnaires.
Home activity feasibility	° < 3 on a 7-point Likert scale, with 6 (0–6 range) representing the highest level of feasibility associated with home activity completion.	° Score of 3 or 4 on a 7-point Likert scale.	° ≥ 5 on a 7-point Likert scale.

Data collected through posttreatment qualitative interviews will be transcribed verbatim and analyzed with NVivo 12.0 software to assist in the identification and manipulation of text segments. As described by Braun et al. [93], an inductive thematic analysis will guide the analytic approach, which involves the identification of salient themes and concepts that arise from interviews. Interviews will be coded independently by two research staff from the study team. Text units will be inductively coded into categories, and then data extracted will be collated to identify potential themes and subthemes. Final themes will be discussed among the research team to identify and resolve any discrepancies until consensus is reached.

To assess enrollment rates, a ratio between participants enrolled versus the number of participants commencing treatment will be calculated. The portion of participants who complete all intervention sessions, including the total mean number of sessions completed, will be estimated as a measurement of participant retention. Additionally, dropout rates and the time-points in which dropouts occurred will be assessed. Questionnaire feasibility will be measured by analyzing the completion rates of measures assessing treatment outcomes and mediators, including the degree of missing data.

To evaluate potential changes in pain outcomes (i.e., pain intensity, pain interference, negative mood, quality of life) and probable mediators (i.e., positive affect, pain acceptance, hopeful thinking, pain self-efficacy), the mean, standard deviation, and range associated with treatment changes in these outcomes will be determined, and 95% CIs will be calculated around each mean. For study outcomes and probable mediators, the minimally important change will be 30% of the baseline score, as this metric has been previously defined as a clinically meaningful change in pain-related symptoms [94]. Using residualized change scores (i.e., regressing the post-intervention scores on the pre-intervention scores for each measure), Pearson’s correlation coefficients will be computed to examine associations between outcomes and intervention target change scores.

## Discussion

Chronic low back pain is a global leader in disability among older adults, rendering geriatric pain management a top health priority [1–3]. Despite the public health impact of cLBP [95], combined with evidence of suboptimal pain management among older adults, interventions that promote positive health outcomes and mitigate pain-associated declines in functioning in this cohort are limited. Likewise, research has been hampered by a predominant emphasis on the exploration of risk factors for chronic pain, while the protective role of resilience factors in pain adaptation has been understudied. Therefore, the present study will add to the existing body of literature by examining the feasibility and acceptability of a 7-session group-based resilience intervention (i.e., ARIAA) in older adults with cLBP.

To date, studies on positive psychological interventions in chronic pain are few; however, preliminary findings support their efficacy. For instance, Hausmann et al. [36] found that in individuals with mild to moderate body pain, an internet-adapted program delivering positive activity skills (i.e., three good things, strengths, gratitude visit, savoring, active-constructive responding, life summary) facilitated reductions in pain 6 months after the intervention. In a subsequent study by the same authors [37], participants randomized to a positive skill building program (aimed at increasing attention to positive events and activities, expressing gratitude and acts of kindness, and increasing engagement in pleasant activities) demonstrated greater improvement in osteoarthritis symptoms, negative affect, and life satisfaction, relative to individuals in a neutral activity program. Muller et al. [34] examined the feasibility and efficacy of an 8-week internet-based positive activity intervention (using tailored positive psychology exercises) for individuals with chronic pain. Improvements were observed in pain intensity and interference, life satisfaction, psychological function, and pain catastrophizing, with several of these effects maintained at 2.5 months posttreatment. A subsequent randomized parallel-group controlled study by Muller et al. [35] found that when compared with a control group practicing mindfulness and recording current life events, the tailored positive activity intervention resulted in significant improvements in pain intensity that were maintained over 3 months. Moreover, in an online randomized controlled trial comparing a positive psychology intervention targeting self-compassion, positive emotions, and optimism with CBT and a waitlist control, Peters et al. [38] found that both active treatments resulted in significant increases in happiness and lower depressive symptoms.

While there is no gold-standard protocol for positive psychology treatments, we have selected activities with known empirical support and supplemented the current protocol with techniques (i.e., cognitive reappraisal, mindfulness) drawn from existing evidence-based therapies for chronic pain (i.e., CBT, ACT) [96, 97]. It is conceivable that a combination of these approaches (e.g., positive psychology plus CBT and ACT) may boost the therapeutic impact of current pain management therapies and could broaden the range of intervention techniques available to patients. An additional innovation of this study is the development of an intervention based upon a mechanistic set of factors (i.e., positive affect, pain acceptance, hope, pain self-efficacy) demonstrating protective effects on pain and psychological functioning from our prior work (K99AG052642) in older adults with cLBP. Further, our intervention supplements current pain management therapies by focusing on individual strengths and abilities and the harnessing of personal resources, rather than alleviating symptoms and deficits. Our 3-month follow-up will allow us to examine the sustainability of potential intervention effects and the feasibility of home activity maintenance. Further, we have selected a group-based format for intervention sessions as this is cost-effective, offers the opportunity for connectedness and social cohesion, and fosters an environment of collaborative learning.

While the study has several strengths, there are some limitations that merit acknowledgment. First, given the pilot nature of this study, our small sample size (*N* = 60) may impact generalizability to the overall population. Although in alignment with the NIH Science of Behavior Change model, we also acknowledge that with a smaller sample our study may be underpowered to examine treatment changes in pain-related outcomes, including putative mediators underlying intervention effects. While these analyses are intended to be exploratory, they will provide preliminary information regarding the potential for success in a future full-scale trial. Nevertheless, caution is warranted in the interpretation of these effects. Second, this study will not include a comparison group which may limit the conclusions that can be drawn regarding the potential effects of the ARIAA intervention, compared with treatment as usual or a waitlist control. However, given the stage of development of ARIAA, this single-arm design is consistent with NIH recommendations for behavioral intervention design and assessment [43]. Third, we will be assessing outcomes at 3 months post-intervention which may be insufficient to address long-term sustainability of treatment effects. And fourth, given recruitment across both clinic and community-based resources, variation in characteristics across these samples may differentially impact treatment outcomes.

In sum, cLBP is a prevalent and burdensome condition that disproportionately affects older adults, resulting in tremendous healthcare and psychological burden. Demonstrating the feasibility and acceptability of the proposed intervention could have a significant impact on the clinical care of older adults with cLBP and may be a critical step toward the advancement of therapeutic modalities aimed at enhancing resilience in older adults with chronic pain.

## Supplementary Information

**Additional file 1.** Post-intervention evaluation questionnaire.

## Data Availability

The datasets used and/or analyzed during the current study are available from the corresponding author on reasonable request.

## Abbreviations

cLBP: Chronic low back pain
CBT: Cognitive behavioral therapy
ACT: Acceptance and commitment therapy
