# Supplementary material for: Adaptability and Resilience in Aging Adults (ARIAA): protocol for a pilot and feasibility study in chronic low back pain
Source: Pilot Feasibility Stud. 2021 Oct 19;7:188. doi: 10.1186/s40814-021-00923-y (PMC8525058; doi:10.1186/s40814-021-00923-y)
Supplement: Supplementary file 1 — Additional file 1. Post-intervention evaluation questionnaire. [file 40814_2021_923_MOESM1_ESM.docx]

Additional File 1

**POST INTERVENTION EVALUATION**

Please follow the instructions below to give the post-treatment interviews. Remember to give the interview in a regular speaking voice and give the participant plenty of time to answer questions. Ask follow-up questions about things you don’t understand or things that would be valuable for making the study better.

**“This part of the question-and-answer interview is very important. We want to know what your experiences were like in this study in your own words. Your comments are going to be used to make the study better. As with all your other data, we will never attach your name to your comments.”**

1. “What were the most important or useful ways to deal with pain that you learned from the groups?” ***Prompts:*** *Describe how it has helped. What kinds of topics do you remember discussing in the group that were helpful for you? What things do you remember learning from the discussion groups?*

________________________________________________________________________________________________________________________________________________________________________________________________________________________________________________________________________________________________________________________________________________________________________________________________________________________________________________________________________________________________________________________________________________

________________________________________________________________________________________

1. “Each week in group, the leaders talked about different topics. Sometimes, you may have felt the information was useful and, other times, not so useful. What were some things covered in the group meetings that were not useful for you?” ***Prompts:*** *Tell me why ____________ was not useful. What parts of the group were your least favorite?* *Did we miss anything? Is there anything else we should talk about?*

__________________________________________________________________________________________________________________________________________________________________________________________________________________________________________________________________________________________________________________________________________________________________________________________________________________________________________________________________________________________________________________________________________

_______________________________________________________________________________________

1. “What was the most useful part about the study?” ***Prompts:*** *Which parts did you like the most? Which parts were your favorite parts? Tell me why ____________ was useful.*

*__________________________________________________________________________________________________________________________________________________________________________________________________________________________________________________________________________________________________________________________________________________________________________________________________________________________________________________________________________________________________________________________________________*

*_______________________________________________________________________________________*

1. “Thinking in a more general way about your life, have you made any meaningful life changes since being in the group? What helped you do that?” ***Prompts:*** *What do you do differently now based on what you’ve learned in the group? What, if anything, do you feel has changed now for you after having completed the program?*

________________________________________________________________________________________________________________________________________________________________________________________________________________________________________________________________________________________________________________________________________________________________________________________________________________________________________________________________________________________________

________________________________________________________________________________

1. “In what ways was the workbook useful or helpful? In what ways was it NOT useful? The CD?”

________________________________________________________________________________________________________________________________________________________________________________________________________________________________________________________________________________________________________________________________________________________________________________________________________________________________________________________________________________________________________________________________________________

________________________________________________________________________________________

1. “How much do you think you will use the materials we gave you? The workbook/CD?”

________________________________________________________________________________________________________________________________________________________________________________________________________________________________________________________________________________________________________________________________________________________________________________________________________________________________________________________________________________________________________________________________________________________________________________________________________________________________________

1. “Dr. Bartley and her researchers are currently looking for ways that they can improve the groups. Were there some parts of the group that you struggled with or had a hard time understanding?”

________________________________________________________________________________________________________________________________________________________________________________________________________________________________________________________________________________________________________________________________________________________________________________________________________________________________________________________________________________________________________________________________________________________________________________________________________________________________________

1. “How did you feel about the home activities? Were they useful? Were there any activities that were easier than others or any that you found difficult? Were there any roadblocks/obstacles to completing the home activities?”

________________________________________________________________________________________________________________________________________________________________________________________________________________________________________________________________________________________________________________________________________________________________________________________________________________________________________________________________________________________________________________________________________________________________________________________________________________________________________

1. “What are your general thoughts regarding the facilitators of the group? Did they present the material in a way that was organized and easy to follow? How you do think they handled the group discussions and questions from each of the members?”

________________________________________________________________________________________________________________________________________________________________________________________________________________________________________________________________________________________________________________________________________________________________________________________________________________________________________________________________________________________________________________________________________________

________________________________________________________________________________________

1. “As a participant, you know a lot about your group now. What do you think we should do differently, or change, to make the groups more effective for the next group of participants?”

________________________________________________________________________________________________________________________________________________________________________________________________________________________________________________________________________________________________________________________________________________________________________________________________________________________________________________________________________________________________________________________________________________

________________________________________________________________________________________

1. “Any other comments you would like to share with us? Is there anything we haven’t asked you about that you think we need to know?”

________________________________________________________________________________________________________________________________________________________________________________________________________________________________________________________________________________________________________________________________________________________________________________________________________________________________________________________________________________________________________________________________________________

________________________________________________________________________________________
